# A genomic analysis of Philadelphia chromosome-negative AML arising in patients with CML

**DOI:** 10.1038/bcj.2016.18

**Published:** 2016-04-08

**Authors:** K Krysiak, M J Christopher, Z L Skidmore, R T Demeter, V Magrini, J Kunisaki, M O'Laughlin, E J Duncavage, C A Miller, B A Ozenberger, M Griffith, L D Wartman, O L Griffith

**Affiliations:** 1 McDonnell Genome Institute, Washington University School of Medicine, St Louis, MO, USA; 2 Department of Medicine, Washington University School of Medicine, St Louis, MO, USA; 3 Department of Pathology and Immunology, Washington University School of Medicine, St Louis, MO, USA; 4 Department of Genetics, Washington University School of Medicine, St Louis, MO, USA; 5 Siteman Cancer Center, Washington University School of Medicine, St Louis, MO, USA

Chronic myelogenous leukemia (CML) is characterized by the Philadelphia chromosome, an acquired clonal abnormality resulting from translocation of chromosomes 9 and 22, and the generation of the *BCR–ABL* fusion oncogene. The development of tyrosine kinase inhibitors (TKIs) has revolutionized the treatment of CML, as TKI therapy leads to inhibition of *BCR–ABL* activity, suppression of the *BCR–ABL*-containing clone and restoration of normal hematopoiesis in the vast majority of cases.

Although TKI therapy is remarkably effective in clearing the *BCR–ABL*-containing clone in CML, it has been associated with the development of non-*BCR–ABL* rearranged (Ph^−^) clonal cytogenetic abnormalities (CCA).^1, 2, 3, 4^ CCA have been observed in 3–10% of cases after complete cytogenetic resolution of the *BCR–ABL* fusion oncogene, occurring 6–18 months after initiation of TKI therapy.^2, 3^ These abnormalities—suggestive of clonal hematopoiesis—are often transient and not associated with CML progression. However, as routine cytogenetic monitoring during TKI therapy is rarely performed, the occurrence of CCA may be underestimated.^2, 4^

Development of Ph^−^ MDS/AML (myelodysplastic syndrome/acute myeloid leukemia) following TKI therapy has been reported to occur infrequently and, in contrast to Ph^−^ CCA, is associated with poor outcomes.^1, 2, 5, 6^ Analysis of two CML patient cohorts treated with TKI therapy reported 2/985 and 3/1701 patients subsequently developed MDS/AML.^1, 5^ Both studies were published with relatively short follow-up, raising the possibility that they may also underestimate the prevalence of Ph^−^ AML.

The relationship between CML and other clonal abnormalities that arise after TKI therapy is unclear, but they have been theorized to result from the unmasking of a premalignant clone that existed before acquisition of *BCR–ABL*.^2, 3, 5, 7^ This scenario raises the possibility of a 'multi-hit' mechanism of CML development in which an early mutation(s) occurs and predisposes the premalignant clone to acquisition (or tolerance) of *BCR–ABL*. A high frequency of somatic mutations was reported in genes associated with myeloid malignancies in Ph^−^ cells of CML patients treated with TKI.^7^ Reports published prior to the TKI era suggested that multiple events are required for CML pathogenesis (postulated by Phil Fialkow and as reviewed by Deininger *et al.*).^4^ In this scenario, one could hypothesize that the early mutation(s) that cooperates with *BCR–ABL* could also cooperate with mutations that lead to AML. Thus, a premalignant clone could increase susceptibility to both leukemias. Alternatively, it is possible that some CML patients harbor an abnormal bone marrow stroma that predisposes them to both the acquisition of *BCR–ABL* and other genetic aberrations (that could lead to MDS or AML), which could arise in distinct founding clones.

Here we report two patients with CML treated with TKIs who achieved complete molecular remission but subsequently developed Ph^−^ AML (Case Synopses). Both patients achieved durable complete molecular remissions before being referred to our center with AML. Consistent with the multi-hit model of leukemogenesis, we hypothesized that both malignancies were clonally related, having arisen from the same premalignant clone, and thus expected the presence of shared variants between the CML and AML.

To test this hypothesis, we performed 'enhanced' exome sequencing (Supplementary Methods) on the AML and CML samples from each patient using skin DNA isolated at the time of AML diagnosis as the 'normal' comparator for each case.^8^ Sequence analysis was performed using the Genome Modeling System.^9^ Mean coverage for filtered variants was over 100 × for all samples with a required minimum coverage of at least 30 ×. For validation, Ion Ampliseq custom panels (Thermo Fisher Scientific, Waltham, MA, USA) were constructed containing each variant (*n*=85). Unfortunately, validation of case 1 variants failed for most amplicons and further analysis could not be performed due to exhaustion of the sample. All case 2 exome variants validated in this manner were found to be present with variant allele frequencies (VAFs) that correlated well with the exome sequencing results (AML, *r*^2^=0.986; CML, *r*^2^=0.955) with a median depth of 2264 × (Figure 1a, Supplementary Methods).

Although trisomy 8 and chromosome 7 abnormalities have been observed in Ph^−^ clones from CML patients that progress to MDS/AML, we observed no copy number variations nor any indication of loss of heterozygosity in these genomes (Supplementary Figures 1*–*3).^10^ Additionally, germline analysis revealed no variants that are known to lead to leukemia susceptibility (Supplementary Results).

Of the 28 somatic variants identified in the case 1 AML, most had a VAF of 30–40% (Figure 1b, Supplementary Table 1). Case 1 CML had 25 variants: 20 at VAFs of 32–53%, 1 on chrX at 84% and 4 variants at VAFs of 10–20%, suggesting the presence of a subclone. We did not identify common variants shared between the CML and the AML samples with the exception of a single four base deletion in a poorly conserved, non-coding region of *EEF1A1* (Supplementary Table 1, Supplementary Figure 4).

Sequence analysis of the case 2 AML sample identified 12 somatic variants, all but one at VAFs of 20–30% in exome data, whereas the CML sample had 21 variants, most at VAFs of 40–50% (Figure 1b, Supplementary Table 2). The somewhat lower VAFs in the AML sample likely reflect the reduced bone marrow involvement by AML at the time of diagnosis (Case Synopses). Following filtering and validation, we observed no common variants between the two leukemias in case 2.

The number of filtered variants found in each sample was comparable with that reported previously.^11, 12, 13^ In each case, most of the variants are non-coding or synonymous, suggesting that they are not pathogenic but reflect pre-existing mutations in the stem cell clone from which the malignancy arose.^12^ We speculated that the single common variant in case 1 might have been the result of a passenger mutation that occurred in early hematopoietic (or mesodermal) development. To test whether it was present in non-malignant hematopoietic cells, we sequenced DNA from sorted peripheral blood T lymphocytes and neutrophils obtained after AML therapy (Case synopses, the patient was in morphological remission but with multilineage dysplasia present in the bone marrow). By Sanger sequencing, the *EEF1A1* deletion was observed in the AML and CML samples but not the T cells (Figure 1c); however, by AmpliSeq analysis, the deletion was present in the sorted T cells at a significant VAF of 1.14% (Supplementary Table 1). We readily detected 9/28 AML variants, including a myeloid malignancy-associated *PRPF8* mutation, in concurrently sorted neutrophils (median VAF 19.4%).^14^ *EEF1A1* was not detected above background (VAF 0.15%) in this cell population. Although the presence of the *EEF1A1* variant in both the AML and CML samples suggests a common clonal origin, the large number of unique variants in each sample, the absence of this variant in the post treatment neutrophils and the lack of other shared variants suggest that each disease arose from a non-malignant clone that diverged early in development. Therefore, it is possible, but unlikely, that the *EEF1A1* deletion, which has not been described previously, contributed to leukemogenesis.

Among the somatic variants in the case 1 AML, we observed an in-frame deletion in *TET2*, a previously reported missense mutation in *PRPF8* and a highly recurrent frameshift deletion in *NPM1* (W288fs, type A), suggesting these variants may act as driver mutations in this case (Supplementary Table 1).^13, 14^ An *NPM1* mutation has been previously described in a CML patient that developed cytogenetically normal AML.^15^ We did not observe the presence of the *NPM1* mutation in the diagnostic CML sample despite 590 × coverage from our enhanced exome sequencing. In the case 2 AML, we observed a missense mutation in *NR2E1* (also known as TLX), a gene found mutated in two other AML cases in the TCGA data set.^13^ It is unclear what role mutations in this gene may have in AML pathogenesis, and there were no other mutations in genes that have been identified as recurrently mutated in AML in this sample. In our CML samples, there were no mutations in genes considered likely drivers of myeloid disease. Still, we cannot rule out the possibility that some of the somatic variants in the CML clones may cooperate with *BCR–ABL* to promote disease.

Our findings suggest that CML and AML arose independently in both patients. It is unlikely that the absence of common variants was the result of limiting sequencing analysis to the 'exome' (in contrast to whole-genome sequencing), as numerous unique variants (*n*=12–28) were observed in each sample. Although both CML and AML are relatively rare diseases, it is possible that in our patients they each arose independently by chance. Alternatively, environmental, stromal or epigenetic factors may predispose some patients to separate hematologic malignancies that arise from distinct cell clones. We also cannot rule out the possibility of TKI-induced secondary malignancy.^3^

In summary, in a subset of CML cases, regression of the Ph^+^ clone does not lead to restoration of normal hematopoiesis. Schmidt *et al.*^7^ reported mutations in leukemia-associated genes shared between Ph^+^ and Ph^−^ clones in a subset of CML patients with CCA, indicating that the Ph^+^ and Ph^−^ clones were derived from a common progenitor that predated the acquisition of *BCR–ABL*. Although the low incidence of secondary malignancy prevents a definitive conclusion that patients being treated for CML are not at increased risk for subsequent MDS/AML, we did not identify the anticipated clear clonal relationship or shared leukemia-associated mutations between the initial CML and subsequent Ph^−^ AML in our patients.

## Figures and Tables

**Figure 1:**
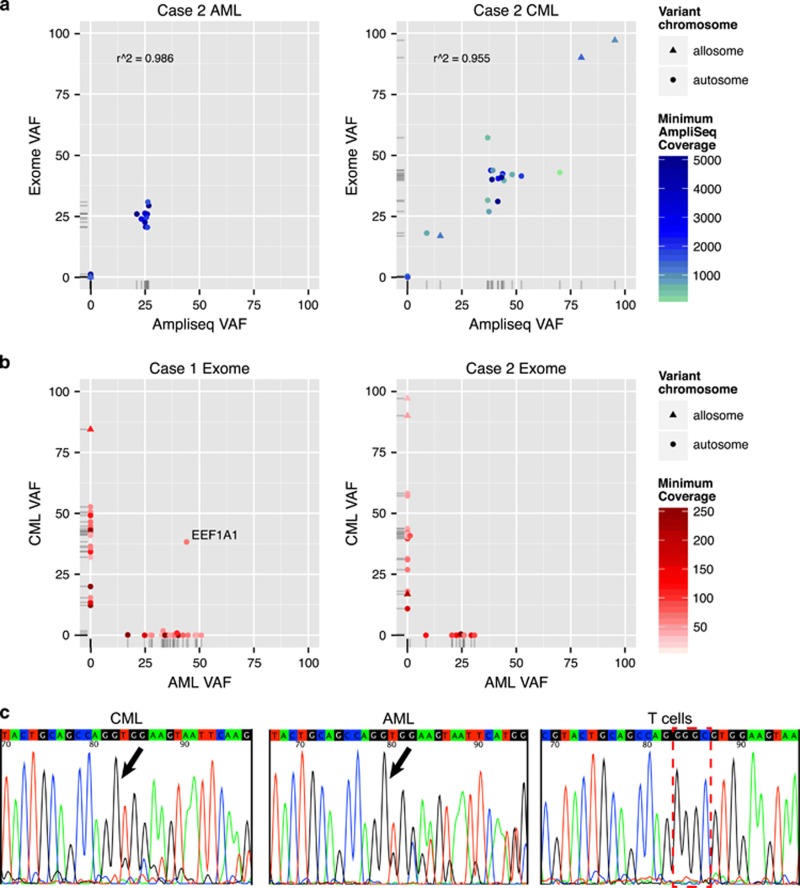
Exome variants in CML samples versus AML samples arising in the same patient. Genomic DNA was isolated from cryopreserved AML bone marrow aspirates, CML bone marrow fixed core and a skin punch biopsy (control) for sequencing. (**a**) Exome (*Y* axis) versus Ampliseq (*X* axis) allele frequencies for case 2 variants that validated by Ampliseq (*N*=29) are plotted for both AML (left panel) and CML (right panel) samples. Pearson's correlation coefficient is shown. (**b**) Filtered exome variants plotted by allele frequency in CML sample (*Y* axis) versus frequency in AML sample (*X* axis) for case 1 (left panel, *N*=52) and case 2 (right panel, *N*=33). (**c**) Sanger sequencing traces showing region of *EEF1A1* 5′ UTR from case 1. CML and AML samples show GGGC deletion in one allele (left and middle panels, arrows) whereas T lymphocytes sorted from a remission sample shows wild-type sequence (right panel, red bracket).
