# Supplementary material for: A genomic analysis of Philadelphia chromosome-negative AML arising in patients with CML
Source: Blood Cancer J. 2016 Apr 8;6(4):e413–. doi: 10.1038/bcj.2016.18 (PMC4855253; doi:10.1038/bcj.2016.18)
Supplement: Supplementary Information [file bcj201618x1.docx]

**Supplemental Materials**

**Case Synopses**

The patients were enrolled in a single-institution, tissue-banking protocol approved by the human studies committee at Washington University. They provided written informed consent for comprehensive sequencing studies, including exome sequencing, in accordance with the Declaration of Helsinki.

*Case 1*

A 72 year old man was diagnosed with chronic phase CML nine years previously and started on imatinib 400mg daily. Family history was notable for a first cousin with AML. Diagnosis cytogenetics were 46, XY, t(9;22)(q34;q11.2) in twenty metaphases. He achieved a complete molecular response on imatinib; when imatinib was subsequently held due to myalgias, he had a molecular relapse with low level, detectable peripheral blood *BCR-ABL* transcripts that resolved after resuming therapy with dasatinib, 100mg daily. He continued to have undetectable *BCR-ABL* transcripts on dasatinib when he was referred to our institution with pancytopenia. Bone marrow biopsy at that time was consistent with acute erythroid leukemia (AML FAB M6) with myelodysplastic changes. Flow cytometry showed 20.6% blasts. The hemodilute bone marrow aspirate differential noted 4% blasts with 80% dysplastic erythroid elements. Sections of the bone marrow core biopsy, which were also suboptimal, showed a markedly hypercellular marrow with marrow cellularity >90%. There was marked erythroid predominance, and erythroids exhibited marked left shift. Cytogenetics were 46, XY and BCR-ABL was negative by both FISH and RT-PCR. He was treated with two cycles of 10 day decitabine on an institutional protocol with no improvement in his blood counts. He was subsequently treated with cladribine, mitoxantrone, and G-CSF with clearance of bone marrow blasts and restoration of normal blood counts, although persistent dysplasia was noted in his bone marrow. The bone marrow aspirate and core showed a mildly hypercellular marrow (approximately 50% cellularity) with multilineage dysplasia. Blasts compromised 1% of marrow cellularity by manual differential and less than 1% by flow cytometry. Cytogenetics were performed and were normal. His peripheral blood BCR-ABL transcripts remained undetectable off TKI therapy. Unfortunately after two months his counts worsened, by three months had ~50% peripheral blasts and after several readmissions for infection he was discharged home on hospice with progressive disease.

*Case 2*

A 54-year-old man was diagnosed with chronic phase CML five years prior to his diagnosis of AML. At diagnosis of CML his white blood cell count was 42,000/ul, hemoglobin 14.5 g/dl, and platelets 683,000/ul. Family history was negative for hematologic malignancy. Bone marrow biopsy was consistent with chronic phase CML, and cytogenetics showed 46, XY, t(9;22)(q34;q11.2) in twenty metaphases. He was treated with imatinib 400mg daily and he achieved a complete hematologic response two months after starting therapy. Seven months after therapy he achieved a major molecular response, and three years after starting therapy with a complete molecular response (CMR). Two years later, while still in a CMR, he developed a macrocytic anemia that did not improve with B12 supplementation or with discontinuation of imatinib. He was found to have detectable BCR-ABL transcripts four months later and was started on dasatinib 75mg daily. Two months after starting dasatinib, he was referred to our institution where a bone marrow biopsy showed an acute myelomonocytic leukemia (AML FAB M4) with 48% blasts and 17% monocytes on the aspirate smear. Cytogenetics could not be performed because of a lack of metaphases. FISH was negative for BCR-ABL, and RT-PCR showed .002% BCR-ABL positivity with respect to β_2_ microglobulin reference transcript. The patient received standard AML induction chemotherapy with cytarabine and idarubicin followed by a second induction due to persistent blasts on a day 14 bone marrow biopsy. He received one round of consolidation chemotherapy before undergoing hematopoietic stem cell transplant from an unrelated donor in first complete remission. He is currently 18 months out from transplant with no evidence of AML. His last bone marrow biopsy showed normal cytogenetics and >95% donor engraftment. Peripheral blood RT-PCR shows a persistent low level of BCR-ABL positivity, which is being followed closely as his immunosuppression is being weaned.

**Supplemental Results**

*Germline analysis*

Analysis of germline variants was performed using the GEMINI workflow pipeline^1^. Germline variants called by SAMtools and VarScan2 were annotated using VEP and integrated with native GEMINI annotations. Variants present within 1000 genomes, dbSNP or the Exome Sequencing Project were excluded from analysis. No variants identified within coding, splice site or UTR regions of genes associated with inherited predisposition to myeloid malignancies^2, 3^ including *TP53*, *RUNX1*, *GATA2*, *CEBPA*, *JAK2* or *DMNT3A* passed manual review. We also investigated shared variants between case 1 and 2. No shared coding variants with a GEMINI predicted ‘HIGH’ or ‘MED’ (medium) impact passed manual review.

*Enhanced exome sequencing of CML-associated genes*

Of the genes previously identified to be mutated by targeted sequencing studies of CML samples (*ASXL1, TET2, TP53, RUNX1, WT1, NRAS, KRAS, IDH1, IDH2, CBLB, CBL, DMNT3A, EZH2, ZRSR2, U2AF1, GATA2*), only *CBLB* and *ZRSR2* were not targeted for higher coverage during exome sequencing by the additional capture reagent spike-in ^4-7^. From this set of genes, only *TET2* was mutated in either of our patients. A somatic *TET2* mutation (p.T1372 in frame deletion) was identified in the AML sample from case 1. However, there was no evidence for this mutation in the CML sample from this patient (**Supplemental Table 1**).

*Evaluation of tumor contamination of the normal samples*

Variants with high VAF in the AML samples were evaluated in matched normal samples taken at the time of AML diagnosis in order to evaluate levels of tumor contamination. AmpliSeq analysis of AML-specific variants in case 1 (27 variants, median coverage 2565x) had a median VAF of 1.62% in the matched normal sample and case 2 (12 variants, median coverage 3270x) had a median VAF of 0.48% in the matched normal sample. The maximum VAF of AML-specific variants in the normal was <3.5% for case 1 and <1% for case 2 indicating it is highly unlikely that key founding clone mutations were missed in the AML samples due to tumor contamination of the corresponding normal samples.

The lack of common variants (both synonymous and nonsynonymous) between AML and CML in each case strongly suggests that each malignancy arose independently in a separate stem cell clone. If there was a shared founding clone, the variants from the founding clone would be present in all of the daughter clones, and thus would be easily identified in our cases, as the tumor purity for all samples was high enough to yield a VAF sufficient for reliable somatic detection (the lowest tumor purity sample was the case 2 AML, which still yielded 20-30% VAFs for the dominant clone).

**Supplemental Methods**

*Enhanced exome sequencing*

Genomic DNA was isolated from cryopreserved AML bone marrow aspirate banked at the time of presentation as well as from formalin fixed, paraffin embedded (FFPE) blocks from the prior CML diagnostic bone marrow core biopsies. To define somatic variants, punch biopsies of patient skin were performed at the time of their AML diagnoses and DNA was isolated from these biopsies, per our institutional protocol, to use as a “normal” comparator. DNA extraction and “enhanced” exome sequencing was performed as previously described ^8-10^. Briefly, dual-indexed (octomer) Illumina libraries were generated using a KAPA HTP sample prep kit, DNA fragmentation using a Covaris E210 DNA sonicator (size range 100-400bp) and prepared using a SciClone instrument. Exome capture was performed using the Roche NimbleGen SeqCap EZ Human Exome Library v3.0 with a custom pool of 120 bp IDT capture probes targeting the coding regions of 264 genes recurrently mutated in AML, the non-coding regions of *WT1* and the promoter region of *TERT* ^8, 9^. This approach typically achieves ~100x median coverage for standard exome target region with deeper coverage of these regions of interest (~400-500x). The Nimblegen SeqCap EZ Exome protocol was used for hybridizations and captured DNA was sequenced on 2 lanes of an Illumina HiSeq 2500 (2x100 bp). A total of 9.2-12.1Gb, of which 6.4-8.2Gb was unique, on-target sequence data was produced for all samples. A minimum 20X depth was achieved for >89.6% of the target sequence and overall mean depth was >79X for all samples.

Preliminary sequencing analysis was performed using the Genome Modeling System^11^. Data were aligned to reference sequence build GRCh37-lite-build37 using BWA-MEM version 0.7.10^12^. Merging and deduplication were performed using picard version 1.113 (https://broadinstitute.github.io/picard/). SNV detection was performed using 5 callers: SAMtools version r982^13^, Somatic Sniper version 1.0.4^14^, VarScan version 2.3.6^15^, Strelka version 1.0.11^16^, and Mutect version 1.1.4. Small indels were detecting using GATK Somatic Indel Detector version 5336^17^, Pindel version 0.5^18^, VarScan, and Strelka. Transcript annotation was performed using Ensembl v74_37.

The sequence data for all tumors and matched normal samples has been deposited in the database of Genotypes and Phenotypes (dbGaP) under accession number: phs000159.

*Variant filters*

Additional filtering strategies were employed to remove technical artifacts, non-somatic events and detected sample cross-contamination. The union of SNV/indel calls from the CML and AML of each case were filtered, removing low quality variants that (A) had less than 30X coverage in all samples counting only reads with a minimum base quality of 20 and minimum mapping quality of 35 (B) had a VAF >15% in the matched normal sample (C) had fewer than 5 supporting reads across both tumor samples (D) were not identified as somatic using a Bayesian classifier with a binomial log likelihood ratio less than 10 (<https://github.com/genome/genome/blob/master/lib/perl/Genome/Model/Tools/Validation/IdentifyOutliers.pm>) and (E) were supported by >10 reads at a >5% VAF in ~15% (5/35) of normal skin enhanced exomes similarly processed. This filtering strategy identified 242 and 121 SNVs and small indels in case 1 and case 2, respectively. Manual review of these variants was performed using Integrative Genomics Viewer (IGV)^19^ to exclude artifacts and miscalls including variants (A) supported only by reads in a single direction (B) caused by adjacent indels (C) called due to insufficient normal coverage (D) occurring within or near long mono- and dinucleotide regions or (E) residing in poor mapping quality regions. Of the filtered variants, 61 and 95 passed manual review, respectively. Regarding the sample cross-contamination, a small degree was observed in both case 1 and case 2 CML samples as low VAF variants at sites of common polymorphism and corresponding to alleles present in samples sequenced around the same time. Putative somatic variants were considered false positives and removed during manual review if found at dbSNP positions or if determined to be germline polymorphisms in the adjacent samples responsible for contamination.

*Copy number/LOH analysis*

Copy number variations (CNVs), were called and plotted from exome data using the R package cn.mops (version 1.8.9) with default parameters. Loss of heterozygosity (LOH) analysis was performed using germline and LOH calls identified via Varscan2 (<http://dkoboldt.github.io/varscan>) using default parameters^15^. Calls were then filtered requiring a normal variant allele frequency (VAF) between 40-60% to identify heterozygous germline variants. The genome was divided into regions using a static window of 100 kb. The absolute difference between normal and tumor VAFs was then calculated and the mean of these differences was obtained for each window. The mean difference was then plotted using the R package ggplot2 to visualize LOH events.

*Ampliseq Validation*

Ampliseq validation was performed on a total of 85 variants (52 case 1 and 33 case 2 variants) not documented in dbSNP or within repetitive regions of the genome (**Supplemental Tables 1 and 2**). Using the Ion AmpliSeq™ Designer version 4.2.4 (www.ampliseq.com), a BED file of targets was submitted for primer design under the standard DNA workflow. Primers were delivered pre-pooled at 2X concentration. In conjunction with the AmpliSeq™ Library Kit 2.0, 50ng of input DNA in 6μl was combined with 10μl of the 2X custom AmpliSeq™ primer pool and 4μl of the 5X Ion AmpliSeq™ HiFi Mix. All reactions were cycled: 99°C for 2 minutes followed by 20 cycles of 99°C for 15 seconds and 60°C for 4 minutes. After amplification, primer sequences were digested by adding 2μl of FuPa Reagent and cycling at 50°C for 10 minutes, 55°C for 10 minutes, and 60°C for 20 minutes. Adapters were ligated onto the samples through the addition of 4μl of Switch Solution, 2μl of Ion Xpress Barcode adapter mix, and 2μl of DNA Ligase. Samples were purified using 45μl of Ampure XP beads (Agencourt/Beckman Coulter).

Libraries were quantified through use of the KAPA Library Quantification Kit for Ion Torrent and diluted to 8pM with Ion Torrent Low TE. All samples (case 1 and case 2) were then pooled together. Template preparation was carried out on the Ion OneTouch 2 instrument in conjunction with the Ion Personal Genome Machine® (PGM™) Template OT2 200 Kit according to revision 3.0 of the corresponding Ion PGM™ Template OT2 200 protocol (publication part number MAN0007221). The amplification reaction consisted of 25μl of nuclease-free water, 500μl of Ion PGM™ Template OT2 200 Reagent Mix, 300μl of 25μl of Ion PGM™ Template OT2 200 PCR Reagent B, 50μl of Ion PGM™ Template OT2 200 Enzyme Mix, 25μl of the 8pM pool of libraries, and 100μl of Ion PGM™ Template OT2 200 Ion Sphere™ Particles (ISPs). Following automated template prep on the OneTouch™ 2, enrichment of template-positive ISPs was done on the Ion OneTouch™ ES instrument. The Ion PGM Sequencing 200 Kit v2 was used for sequencing on the Ion Torrent PGM™. Sequence was generated on one 318 version 2 chip. Torrent Suite version 4.4.3 was used to provide preliminary coverage analysis against the custom target regions.

Case 1 and case 2 normal and tumor samples were pooled for sequencing and all 85 variants were analyzed for both samples, with somatic variants predicted for one case serving as an internal control for the other. Additional AmpliSeq was performed on post-AML remission sorted T lymphocytes and neutrophils for case 1, which were processed separately. The majority of amplicons from both case 1 tumor samples failed due to technical reasons and had insufficient material to be rerun. Of the 33 variants identified by exome sequencing of case 2, two failed to be targeted by this strategy resulting in no coverage at these sites in the case 1 normal and all case 2 samples. Data was aligned to reference sequence build GRCh37-lite-build37 using the same methods described above for exome sequencing except without deduplication. Mean depth ranged from 4,126-4,719x across case 2 normal, AML and CML samples. To be considered as validated, variants were required to have at least 20x coverage from reads with a minimum base quality of 20 and a minimum mapping quality of 35 as well as >1% variant allele frequency in the sample in which it was originally identified. In order to eliminate variants identified as a result of sample cross-talk from our pooling strategy, variants were determined to have failed validation if they were identified as having >5% total VAF in all samples from the opposing case. In total, 2 variants from case 2 failed validation based on these criteria and 2 were not successfully targeted and therefore could not be confirmed, resulting in 29/33 case 2 variants as validated.

*Validation of EEF1A1 deletion*

Genomic DNA from the CML and AML library preps was used as the template for PCR using primers amplifying the region of interest in the *EE1FA1* 5’ UTR (left primer 5’-AGTGCAGTAGTCGCCGTGAA-3’, right primer 5’-AGGCCTCAACTCAAGCACGA). PCR product was purified using NucleoSpin Gel and PCR Clean-up kit (Macherey-Nagel, Bethlehem, PA) and sequenced from both directions using the same PCR primers on an Applied Biosystems Model 3730 DNA sequencer at the Protein and Nucleic Acid Chemistry Laboratory core facility at Washington University School of Medicine. To test whether the deletion was present in normal hematopoietic cells, T lymphocytes and neutrophils from a cryopreserved remission sample banked 172 days after AML diagnosis were sorted using flow cytometry and anti-human CD45 (PerCP-Cy5.5, BD Biosciences, San Jose, CA), CD15 (FITC, BD Biosciences, San Diego, CA) and CD3 (eFlour 450, Clone: OKT3, eBioscience, San Diego, CA) antibodies. Genomic DNA was isolated using DNeasy Blood and Tissue Kit (Qiagen, Valencia, CA) and analyzed by PCR and sequencing as above.

**Supplemental Figures**

**Supplemental Figure 1.** **Copy number plots for case 1 acute myeloid leukemia**

Copy number variations between case 1 normal skin and the diagnostic AML sample were plotted as calculated using cn.mops (1.8.9). Consistent with diagnostic AML bone marrow cytogenetics, no large-scale copy number changes were observed using exome sequencing data.

**
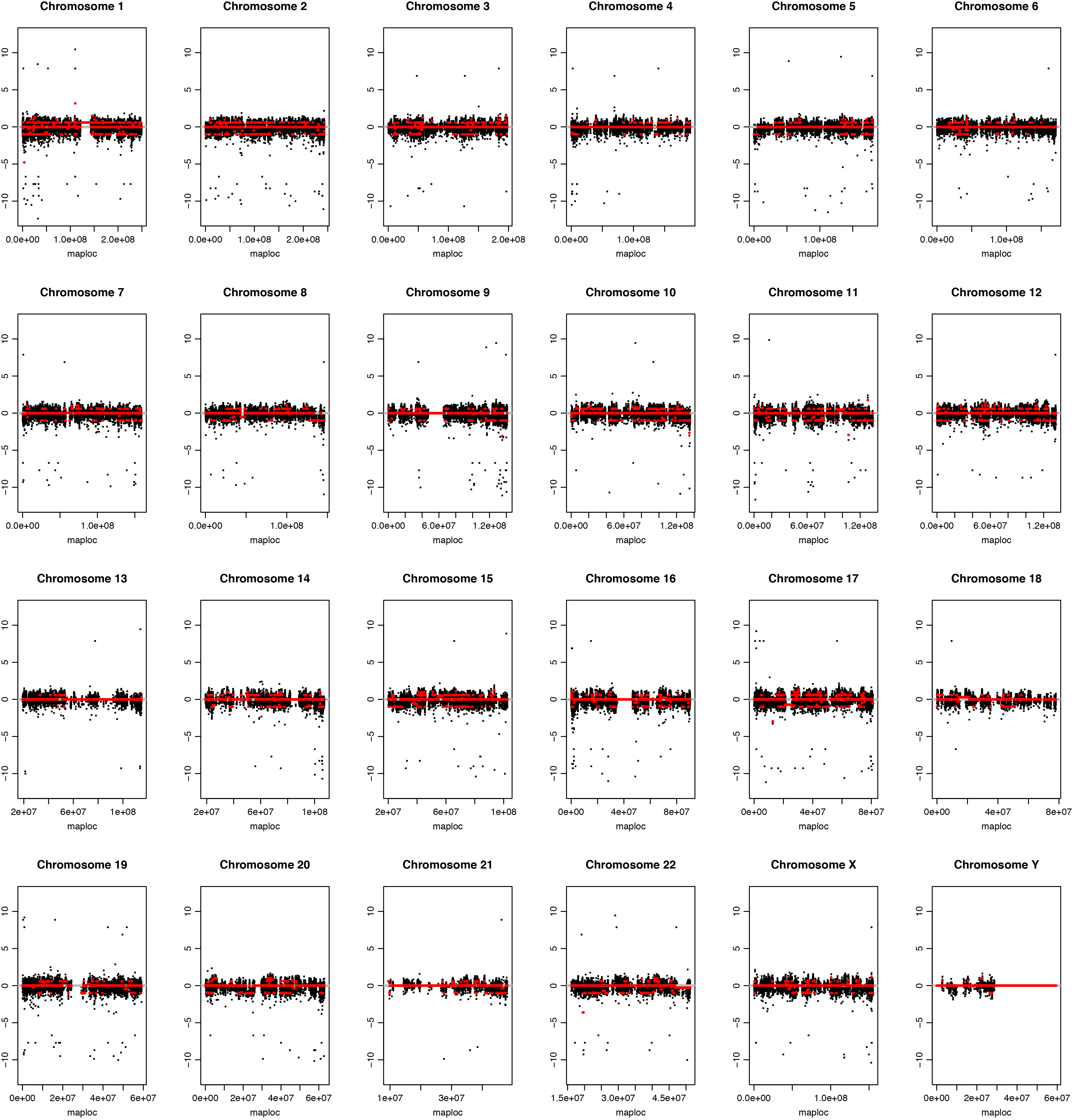
**

**Supplemental Figure 2.** **Copy number plots for case 2 acute myeloid leukemia**

Copy number variations between case 2 normal skin and a diagnostic AML sample were plotted as calculated using cn.mops (1.8.9). No large-scale copy number changes were observed using exome sequencing data.

**
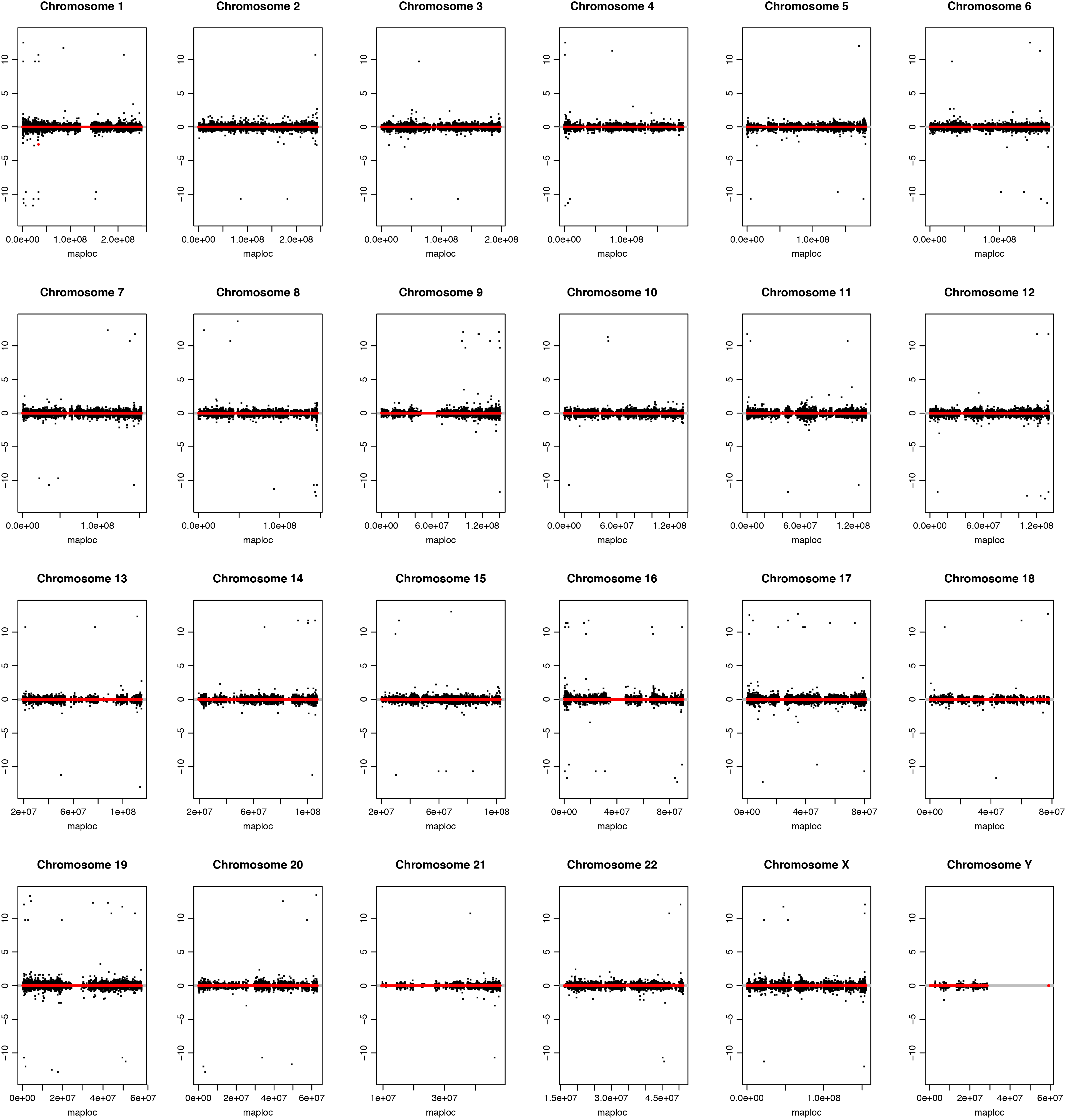
**

**Supplemental Figure 3.** **Loss of heterozygosity plots for each patient**

Positions identified as heterozygous, defined as having a variant allele frequency between 40-60%, in each patient’s normal sample were selected. These positions were binned in static windows of 100kb, and the absolute difference between tumor and normal VAFs was calculated. The average difference within each window was then plotted. As expected for male patients, heterozygous sites were not identified on chromosome X in the normal samples for comparison to the tumor samples.

**Supplemental Figure 4.** **The *EEF1A1* variant occurs in a poorly conserved, non-coding region of the gene**

Screenshots from the USCS Genome Browser displaying the reference sequence (GRCh37/hg19), UCSC and Ensembl transcripts, and mammalian conservation for the *EEF1A1* locus are shown. The two views display A) all transcripts and B) the region immediately surrounding the deletion. The sequence removed by the 4 bp *EEF1A1* deletion variant is indicated in blue.

**
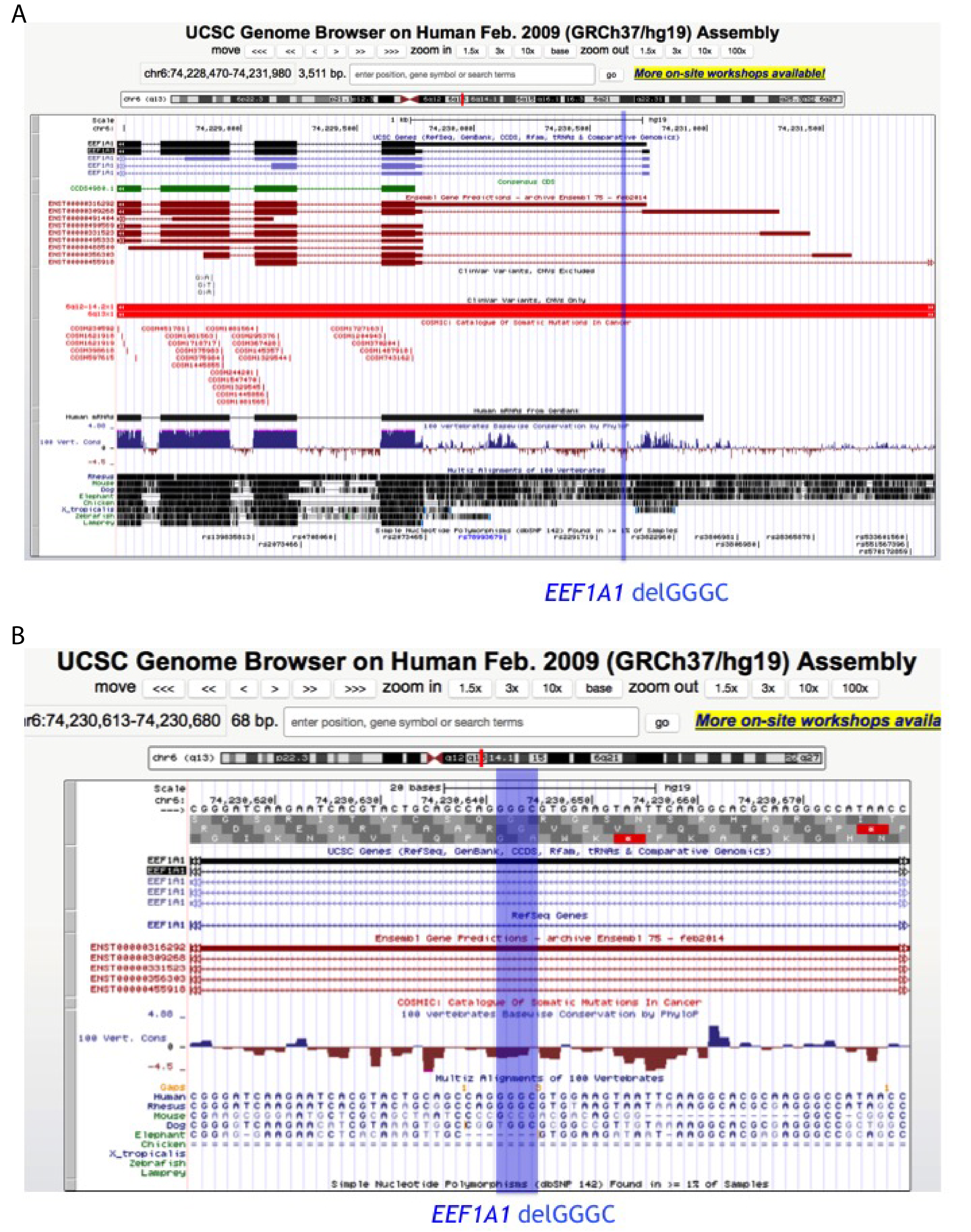
**

**References**

1. Paila U, Chapman BA, Kirchner R, Quinlan AR. GEMINI: integrative exploration of genetic variation and genome annotations. *PLoS computational biology* 2013; **9**(7)**:** e1003153.

2. Cazzola M, Della Porta MG, Malcovati L. The genetic basis of myelodysplasia and its clinical relevance. *Blood* 2013 Dec 12; **122**(25)**:** 4021-4034.

3. Godley LA. Inherited predisposition to acute myeloid leukemia. *Semin Hematol* 2014 Oct; **51**(4)**:** 306-321.

4. Grossmann V, Kohlmann A, Zenger M, Schindela S, Eder C, Weissmann S*, et al.* A deep-sequencing study of chronic myeloid leukemia patients in blast crisis (BC-CML) detects mutations in 76.9% of cases. *Leukemia* 2011 Mar; **25**(3)**:** 557-560.

5. Makishima H, Jankowska AM, McDevitt MA, O'Keefe C, Dujardin S, Cazzolli H*, et al.* CBL, CBLB, TET2, ASXL1, and IDH1/2 mutations and additional chromosomal aberrations constitute molecular events in chronic myelogenous leukemia. *Blood* 2011 May 26; **117**(21)**:** e198-206.

6. Schmidt M, Rinke J, Schafer V, Schnittger S, Kohlmann A, Obstfelder E*, et al.* Molecular-defined clonal evolution in patients with chronic myeloid leukemia independent of the BCR-ABL status. *Leukemia* 2014 Dec; **28**(12)**:** 2292-2299.

7. Zhang SJ, Ma LY, Huang QH, Li G, Gu BW, Gao XD*, et al.* Gain-of-function mutation of GATA-2 in acute myeloid transformation of chronic myeloid leukemia. *Proceedings of the National Academy of Sciences of the United States of America* 2008 Feb 12; **105**(6)**:** 2076-2081.

8. Cancer Genome Atlas Research N. Genomic and epigenomic landscapes of adult de novo acute myeloid leukemia. *The New England journal of medicine* 2013 May 30; **368**(22)**:** 2059-2074.

9. Klco JM, Miller CA, Griffith M, Petti A, Spencer DH, Ketkar-Kulkarni S*, et al.* Association Between Mutation Clearance After Induction Therapy and Outcomes in Acute Myeloid Leukemia. *Jama* 2015 Aug 25; **314**(8)**:** 811-822.

10. Ellis MJ, Ding L, Shen D, Luo J, Suman VJ, Wallis JW*, et al.* Whole-genome analysis informs breast cancer response to aromatase inhibition. *Nature* 2012 Jun 21; **486**(7403)**:** 353-360.

11. Griffith M, Griffith OL, Smith SM, Ramu A, Callaway MB, Brummett AM*, et al.* Genome Modeling System: A Knowledge Management Platform for Genomics. *PLoS computational biology* 2015 Jul; **11**(7)**:** e1004274.

12. Li H, Durbin R. Fast and accurate short read alignment with Burrows-Wheeler transform. *Bioinformatics* 2009 Jul 15; **25**(14)**:** 1754-1760.

13. Li H, Handsaker B, Wysoker A, Fennell T, Ruan J, Homer N*, et al.* The Sequence Alignment/Map format and SAMtools. *Bioinformatics* 2009 Aug 15; **25**(16)**:** 2078-2079.

14. Larson DE, Harris CC, Chen K, Koboldt DC, Abbott TE, Dooling DJ*, et al.* SomaticSniper: identification of somatic point mutations in whole genome sequencing data. *Bioinformatics* 2012 Feb 1; **28**(3)**:** 311-317.

15. Koboldt DC, Zhang Q, Larson DE, Shen D, McLellan MD, Lin L*, et al.* VarScan 2: somatic mutation and copy number alteration discovery in cancer by exome sequencing. *Genome research* 2012 Mar; **22**(3)**:** 568-576.

16. Saunders CT, Wong WS, Swamy S, Becq J, Murray LJ, Cheetham RK. Strelka: accurate somatic small-variant calling from sequenced tumor-normal sample pairs. *Bioinformatics* 2012 Jul 15; **28**(14)**:** 1811-1817.

17. McKenna A, Hanna M, Banks E, Sivachenko A, Cibulskis K, Kernytsky A*, et al.* The Genome Analysis Toolkit: a MapReduce framework for analyzing next-generation DNA sequencing data. *Genome research* 2010 Sep; **20**(9)**:** 1297-1303.

18. Ye K, Schulz MH, Long Q, Apweiler R, Ning Z. Pindel: a pattern growth approach to detect break points of large deletions and medium sized insertions from paired-end short reads. *Bioinformatics* 2009 Nov 1; **25**(21)**:** 2865-2871.

19. Robinson JT, Thorvaldsdottir H, Winckler W, Guttman M, Lander ES, Getz G*, et al.* Integrative genomics viewer. *Nature biotechnology* 2011 Jan; **29**(1)**:** 24-26.
