# Supplementary material for: A genomic analysis of Philadelphia chromosome-negative AML arising in patients with CML
Source: Blood Cancer J. 2016 Apr 8;6(4):e413–. doi: 10.1038/bcj.2016.18 (PMC4855253; doi:10.1038/bcj.2016.18)
Supplement: Supplementary Table Legends [file bcj201618x4.docx]

**Supplemental Table 1: Case 1 somatic variants**
Case 1 variants identified by exome sequencing of the chronic myeloid leukemia and subsequent acute myeloid leukemia. Variants were called by MuTect (v1.1.4), Strelka (v1.0.11), SAMtools (r982), Somatic Sniper (v1.0.4), VarScan (v2.3.6), GATK Somatic Indel Detector (v5336) against the GRCh37 human reference genome. Annotations were performed by the MGI Annotator against Ensembl version v74_37. Read counts and variant allele frequency (VAF) calculations for these variants in exome and AmpliSeq data were determined using bam-readcount (v0.7) requiring a minimum base and mapping quality of 20 for reads to be counted. Variants were considered validated by AmpliSeq if they had at least 20x coverage and a VAF > 0.1% in the sample where they were initially identified as well as <5% VAF in the unrelated Case 2 samples. Read counts are also provided for Ampliseq performed on T lymphocytes (T cell) and neutrophils (PMNs) sorted from a post-AML remission peripheral blood sample. See **Supplemental Methods** for more detail.

**Supplemental Table 2: Case 2 somatic variants**
Case 2 variants identified by exome sequencing of the chronic myeloid leukemia and subsequent acute myeloid leukemia. Variants were called by MuTect (v1.1.4), Strelka (v1.0.11), SAMtools (r982), Somatic Sniper (v1.0.4), VarScan (v2.3.6), GATK Somatic Indel Detector (v5336) against the GRCh37 human reference genome. Annotations were performed by the MGI Annotator against Ensembl version v74_37. Read counts and variant allele frequency (VAF) calculations for these variants in exome and AmpliSeq data were determined using bam-readcount (v0.7) requiring a minimum base and mapping quality of 20 for reads to be counted. Variants were considered validated by AmpliSeq if they had at least 20x coverage and a VAF > 0.1% in the sample where they were initially identified as well as <5% VAF in the unrelated Case 1 samples. See **Supplemental Methods** for more detail.
